# Beyond the skin: immunological profiles and infectious complications in ALOX12B-associated autosomal recessive congenital ichthyosis

**DOI:** 10.3389/fimmu.2025.1662858

**Published:** 2025-11-19

**Authors:** Asena Pinar Sefer, Mehmet Cihangir Catak, Isa An, Necmiye Keser Ozturk, Leyla Baykal Selcuk, Oguz Salih Dincer, Mehdi Benamar, Feven Getachew, Klaus Schmitz-Abe, Pankaj B. Agrawal, Feyza Bayram Catak, Baran Erman, Sevgi Bilgic Eltan, Elif Karakoc Aydiner, Ahmet Ozen, Talal Chatila, Safa Baris

**Affiliations:** 1 Division of Allergy and Immunology, Department of Pediatrics, School of Medicine, Recep Tayyip Erdogan University, Rize, Türkiye; 2 Department of Pediatric Allergy and Immunology, Faculty of Medicine, Marmara University, Istanbul, Türkiye; 3 Istanbul Jeffrey Modell Diagnostic Center for Primary Immunodeficiency Diseases, Istanbul, Türkiye; 4 Isil Berat Barlan Center for Translational Medicine, Istanbul, Türkiye; 5 Immune Deficiency Research and Application Center, European Academy of Allergy and Clinical Immunology, Marmara University Hospital Center of Excellence, Istanbul, Türkiye; 6 Department of Dermatology, Sanliurfa Research and Training Hospital, Sanliurfa, Türkiye; 7 Department of Dermatology, School of Medicine, Karadeniz Technical University, Trabzon, Türkiye; 8 Division of Hematology and Oncology, Department of Pediatrics, School of Medicine, Recep Tayyip Erdogan University, Rize, Türkiye; 9 Division of Immunology, Department of Pediatrics, Harvard Medical School, Boston Children’s Hospital, Boston, MA, United States; 10 Division of Neonatology, Department of Pediatrics, University of Miami Miller School of Medicine and Holtz Children’s Hospital, Jackson Health System, Miami, FL, United States; 11 Institute of Child Health, Hacettepe University, Ankara, Türkiye; 12 Can Sucak Research Laboratory for Translational Immunology, Hacettepe University, Ankara, Türkiye

**Keywords:** ALOX12B, ARCI, autosomal recessive congenital ichthyosis, Hyper-IgE, immunodeficiency, immune dysregulation

## Abstract

**Background:**

Pathogenic variants in *ALOX12B*, a crucial enzyme involved in epidermal lipid processing, are among the most common causes of autosomal recessive congenital ichthyosis (ARCI). Although traditionally considered a cutaneous disorder, the systemic immunological implications of ALOX12B deficiency remain poorly understood.

**Objectives:**

We aimed to broaden the dermatologic and immunologic spectrum of ALOX12B-associated ARCI by characterizing the clinical, immunologic, and genetic features of six patients from three consanguineous families.

**Methods:**

This prospective study included six patients with ALOX12B-associated ARCI identified through whole-exome sequencing. Detailed dermatological evaluations, infection histories, immunoglobulin profiles, lymphocyte subset analyses, and vaccine response assessments were performed.

**Results:**

All patients exhibited early-onset generalized ichthyosis, ranging from delayed-onset lamellar ichthyosis to collodion membrane presentations accompanied by nonbullous erythroderma. Two distinct biallelic *ALOX12B* variants were identified: a novel p.Thr383Lys and the known p.Cys544Arg. Several patients demonstrated recurrent bacterial or fungal infections (n = 5), markedly elevated serum IgE levels (n = 4), and isolated abnormalities in vaccine responsiveness (n = 2). Lymphocyte counts and other immunoglobulin classes were generally preserved; however, decreased IgG levels were observed in one patient (P3.1). Intravenous immunoglobulin replacement therapy reduced the frequency of infections in patients (P1.1 and P1.2).

**Conclusions:**

Our findings suggest that ALOX12B-related ARCI may involve secondary immune dysregulation, driven by chronic compromise of the epidermal barrier. An immunologic evaluation is warranted in selected cases, particularly those with a history of susceptibility to infections. Multidisciplinary care, encompassing dermatology, immunology, and genetics, is crucial for achieving optimal outcomes in ARCI.

## Introduction

Inherited ichthyoses represent a clinically and genetically diverse group of cornification disorders, primarily characterized by widespread scaling and varying degrees of erythroderma (1). Among these, autosomal recessive congenital ichthyosis (ARCI) represents a major subgroup, categorized into lamellar ichthyosis (LI), non-bullous congenital ichthyosiform erythroderma (CIE), and harlequin ichthyosis (HI). While LI is typically characterized by large, dark, plate-like scales with minimal erythema, and may be accompanied by ectropion, eclabium, and nail abnormalities, CIE presents fine white scaling and more pronounced erythema (2). A collodion membrane (CM) is often present at birth in both LI and CIE, and its resolution during the first weeks of life may reveal varying degrees of persistent ichthyosis or, in some cases, a self-improving phenotype (3). Pathogenic variants in more than a dozen genes have been identified as contributing to ARCI. The most commonly mutated genes include *TGM1* (transglutaminase 1), *ABCA12* (ATP-binding cassette sub-family A member 12), *ALOX12B* (arachidonate 12-lipoxygenase, 12R type), *ALOXE3* (arachidonate lipoxygenase 3), *CYP4F22* (Cytochrome P450 4F22), *NIPAL4* (ichthyin), *PNPLA1* (Patatin-like phospholipase domain-containing protein 1), *CERS3* (Ceramide synthase-3), and *SLC27A4* (Solute Carrier Family 27 Member 4) (4).

Pathogenic variants in *ALOX12B*, a gene encoding the 12R-lipoxygenase enzyme involved in epidermal lipid metabolism and skin barrier formation, are among the most commonly implicated genetic defects in ARCI. To date, 162 pathogenic variants have been identified. These variants can lead to variable phenotypic severity, ranging from mild, generalized scaling with little or no erythema to more classic presentations of LI or CIE (5). Disruption of the enzyme’s catalytic C-terminal domain results in a non-functional protein, impairing acylceramide synthesis and compromising epidermal barrier integrity (6). Interestingly, variants are frequently associated with self-improving collodion ichthyosis (SICI), particularly in specific populations (7). Some reports note recurrent infections in ARCI patients, possibly secondary to chronic barrier defects (8). Despite growing knowledge of its dermatological manifestations, there is a remarkable lack of studies evaluating the immunological profile of patients with *ALOX12B* mutations. In particular, it is unclear whether the skin barrier defect can drive broader immune dysregulation, for example, via chronic inflammation or allergic sensitization pathways, or whether immunologic abnormalities contribute to disease severity.

In this study, we describe six patients referred to our center due to recurrent cutaneous bacterial and fungal infections and/or markedly elevated serum IgE levels, in whom biallelic pathogenic *ALOX12B* variants were subsequently identified. Through clinical, immunological, and genetic evaluation, we aim to investigate the potential connection between skin barrier impairment and immune dysregulation in this subset of patients.

## Materials and methods

The study included six patients diagnosed with *ALOX12B* congenital ichthyosis, characterized by unusual immunological features. The Marmara University Faculty of Medicine Ethics Committee approved the study protocol. Before initiating any study procedures, written informed consent was obtained from the legal guardians of all patients. Written informed consent for participation and publication of clinical photographs was obtained from all patients or their legal guardians, and all studies were conducted in accordance with the principles outlined in the Declaration of Helsinki.

The genetic diagnosis was made through whole-exome sequencing (WES) and confirmed by Sanger sequencing. Genomic DNA was extracted from peripheral blood using standard kits. WES was performed using Agilent SureSelect Human All Exon or IDT xGen Exome capture kits, sequenced on Illumina NovaSeq 6000 or BGISeq-500 platforms with 75 bp paired-end reads. Coverage depth ensured ≥85% of bases at ≥20×. Reads were aligned to hg19 using BWA-MEM, with variant calling by GATK. Annotation was performed using VEP and ANNOVAR, with filtering via GEMINI. Variants were retained if depth >20, GQ >15, MAF <0.01 (1K Genomes, gnomAD), CADD Phred >5, and VAF >0.25. Synonymous variants were excluded. Pathogenicity was assessed using SIFT, PolyPhen, MutationTaster, and GERP conservation scores, and interpreted in accordance with ACMG guidelines. Candidate variants were confirmed by Sanger sequencing using Big Dye Terminator v1.1 (Applied Biosystems).

The structural and evolutionary analysis of the identified variant was performed using a combination of sequence- and structure-based tools. The corresponding protein sequence was first analyzed by BLAST against the NCBI Reference Sequence (RefSeq) protein database to identify homologous sequences. The resulting sequences were exported in FASTA format and aligned using Clustal Omega v1.2.4 (9–11). The multiple sequence alignment was visualized with ESPript 3.0 (12) to assess residue conservation. The predicted three-dimensional structure of the protein was obtained from the AlphaFold Protein Structure Database (13, 14). Structural visualization and analyses were performed using UCSF ChimeraX v1.10.1 (15).

Details are provided in the **Supplementary file**. Variants were interpreted in relation to clinical findings, and additional immunodeficiency-related genes were screened to rule out other primary immunodeficiencies (PIDs).

Their demographic and clinical data (age of onset, age at diagnosis, duration of follow-up, family history, dermatologicial manifestations, allergic symptoms, previous infections, systemic involvement, diagnostic examination, treatments, and clinical course) were obtained from their medical records. Growth parameters and developmental status were also assessed.

Serum immunoglobulin levels and vaccine responses were evaluated in all patients. Serum total IgE levels were quantified using an enzyme-linked immunosorbent assay (ELISA). Age-adjusted reference intervals were applied according to the manufacturer’s instructions. Hyper-IgE was defined as serum IgE levels exceeding 1000 IU/mL, irrespective of age, consistent with previously published criteria for elevated IgE in population surveys and PID cohorts (16, 17). This threshold distinguishes moderate IgE elevation secondary to atopy from markedly increased levels typically observed in Hyper-IgE Syndrome (HIES), which is defined as greater than 2000 IU/mL. Peripheral lymphocyte subgroup analyses were performed using flow cytometry and compared to age-matched reference data, as previously described (18–20). Peripheral blood mononuclear cells were isolated and stained with a multicolor antibody panel for flow cytometry (Navios EX, Beckman Coulter). The following markers were used: CD3, CD4, CD8, CD45, CD45RA, CCR7, CD19, CD27, IgD, CD21, CD38, and CD25 (see full antibody list in **Supplementary Table S1**). After incubation and red cell lysis, cells were acquired and analyzed with Kaluza v2.1 software. For advanced evaluation, lymphocyte subset profiles of the patients were analyzed and compared with age-matched healthy controls (HCs).

Statistical analyses were conducted using paired t-tests or Wilcoxon matched-pairs tests, as appropriate. A two-tailed p value of <0.05 was considered statistically significant. Figures were created using Adobe Illustrator 25.2.1 (Adobe Inc., San Jose, California) and GraphPad Prism version 9.0.0 (GraphPad Software Inc., San Diego, California).

## Results

### Patient cohort and demographics

**Figure 1:**
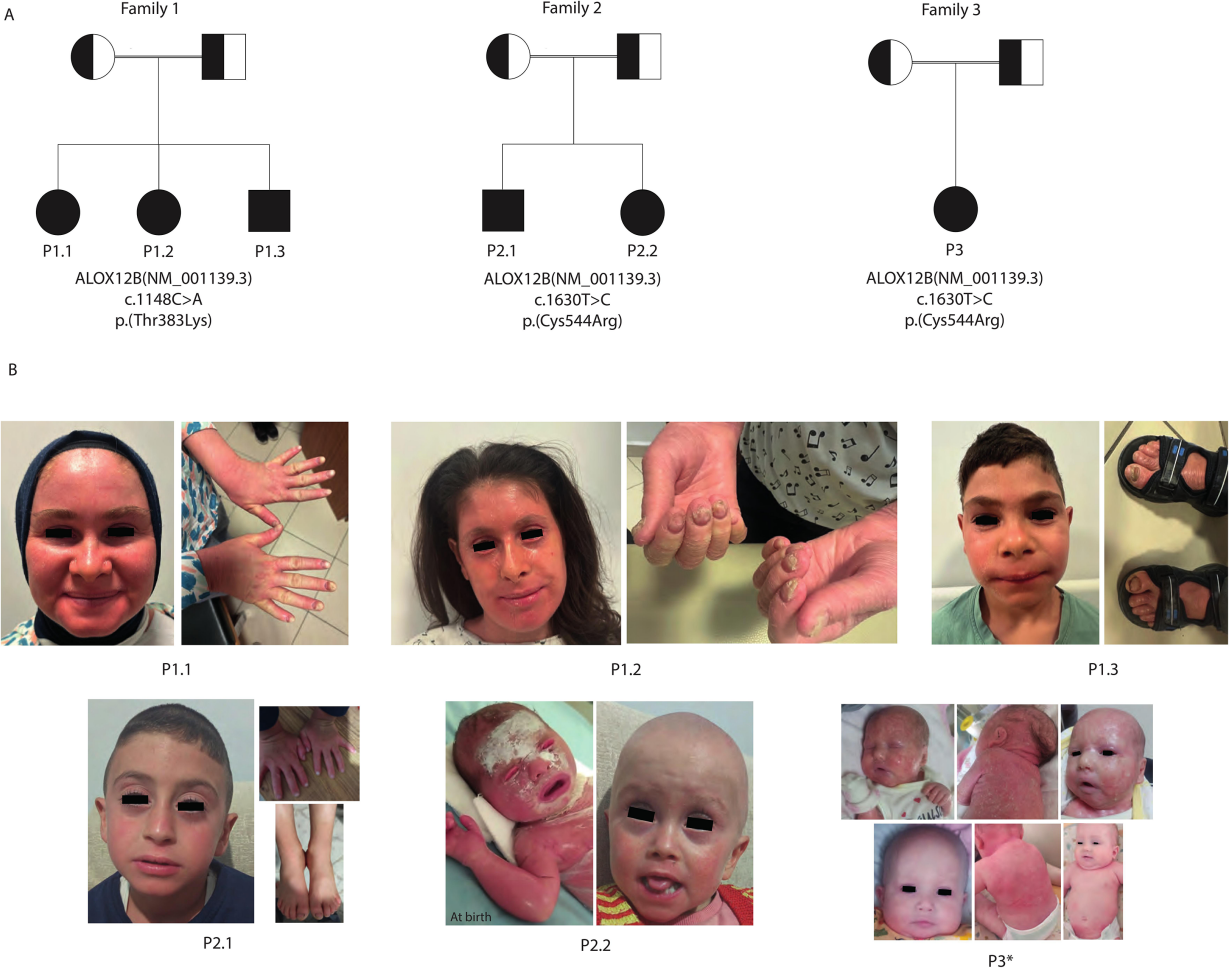
Pedigrees and representative dermatological findings of patients. **(A)** Pedigrees of the three consanguineous families (Families 1–3) illustrate autosomal recessive inheritance of *ALOX12B* variants. Squares represent male individuals, circles represent female individuals, filled symbols denote affected patients, and double horizontal lines indicate parental consanguinity. **(B)** Clinical photographs of affected patients demonstrating hallmark cutaneous features.. P1.1: Facial erythema with fine scaling and onychodystrophy. P1.2: Generalized facial erythema with fine white scaling, ectropion, periungual hyperkeratosis, and severe onychodystrophy. P1.3: Mild facial erythema with limited scaling and toenail onychodystrophy. P2.1: Mild generalized erythema with fine scaling and onychodystrophy. P2.2: Neonatal collodion membrane at birth, evolving into fine scaling, mild erythema during infancy, and alopecia totalis. P3: Self-improving collodion ichthyosis (SICI) phenotype showing severe neonatal collodion presentation with subsequent marked improvement and near-normalization of skin by infancy and alopecia totalis. These findings illustrate hallmark cutaneous features of ALOX12B deficiency, including collodion baby presentation, fine light-colored scaling with variable erythema, occasional nail changes (onychodystrophy), absence of pronounced keratoderma, and a tendency toward milder or self-improving disease course. *: Photos of P3 before (top row) and after (bottom row) systemic retinoic acid therapy.

This study included six patients from three unrelated consanguineous Turkish families; all diagnosed with ARCI due to *ALOX12B* variants. In Family 1, there were three affected siblings: P1.1, a 17-year-old female; P1.2, a 14-year-old female; and P1.3, a 6-year-old male, each carrying the same homozygous c.1148C>A (p.Thr383Lys) *ALOX12B* variant. Family 2 contained two affected siblings, P2.1, a 6-year-old male, and P2.2, a 4-year-old female, while Family 3 had one affected child, P3.1, a 1-year-old female; all were homozygous for the c.1630T>C (p.Cys544Arg) *ALOX12B* variant. All parents were first-degree cousins and confirmed to be heterozygous for the respective variants (**Table 1**, **Figure 1A**).

**Table 1:** The demographical, clinical, and genetic features of ALOX12B patients.

Family	Family 1			Family 2		Family 3
Subject	P1.1	P1.2	P1.3	P2.1	P2.2	P3
Sex	Female	Female	Male	Male	Female	Female
Current age (y)	17	14	8	6	3	1
Ichthyosis presentation at birth	No	No	No	Marked scaling and erythema	CM	CM
Consanguinity	Yes	Yes	Yes	Yes	Yes	Yes
Clinical type	LI	LI	LI	CIE	CIE	CIE
Skin phenotype	Yes	Yes	Yes	Yes	Yes	Yes
Hair involvement	No	No	No	No	Alopecia	Alopecia
Nail involvement	Onychodystrophy	Onychodystrophy	Onychodystrophy	Onychodystrophy	No	No
FTT	No	Yes	No	No	Yes	No
Allergic manifestations	Asthma, AR	Asthma, AR	No	No	Asthma	No
Other clinical findings	–	–	**-**	**-**	Chronic lung disease	**-**
*ALOX12B* Gene Variant (NM_001139.3)	c.1148C>A p.(Thr383Lys)	c.1148C>A p.(Thr383Lys)	c.1148C>A p.(Thr383Lys)	c.1630T>C p.(Cys544Arg)	c.1630T>C p.(Cys544Arg)	c.1630T>C p.(Cys544Arg)
Type of mutation	Missense	Missense	Missense	Missense	Missense	Missense
Zygosity	Homozygous	Homozygous	Homozygous	Homozygous	Homozygous	Homozygous
Previously reported	Novel	Novel	Novel	Yes	Yes	Yes
ACGM classification	Likely pathogenic	Likely pathogenic	Likely pathogenic	Pathogenic	Pathogenic	Pathogenic

A, Adenine; ACMG; American College of Medical Genetics and Genomics; AR, Allergic rhinitis; Arg, Arginine, c., Coding DNA change; C, Cytosine; CIE, Congenital ichthyosiform erythroderma; CM, Collodion membrane; cys, Cysteine; FTT, Failure to thrive; LI, Lamellar ichthyosis; Lys, Lysine; NM, Nucleotide Messenger RNA sequence; P, Patient; p., Protein-level amino acid substitution; T, Thymine; Thr, Threonine; y, years.

### Dermatological findings

All six affected individuals presented with early-onset generalized ichthyosis; however, the clinical subtype, neonatal presentation, and disease progression varied both across and within families. The three siblings in Family 1 (P1.1, P1.2, and P1.3) exhibited a phenotype consistent with LI, characterized by large, dark, plate-like scales and underlying erythema. Their skin appeared unremarkable during the neonatal period, but the ichthyosis became clinically significant after the first year of life, indicating a delayed-onset LI pattern.

In contrast, patients from Families 2 and 3 demonstrated clinical features more consistent with nonbullous CIE. Specifically, P2.2 and P3 were born with a classic collodion membrane, whereas P2.1 exhibited marked scaling and erythema, but without a collodion phenotype. Although the membrane resolved in P2.2 and P3.1 during early infancy, persistent erythroderma and generalized scaling developed subsequently. P2.1 followed a similar disease trajectory with chronic inflammation and hyperkeratosis. Importantly, P2.1 exhibited a milder skin involvement and no alopecia, in contrast to his sibling P2.2 and P3, who had total congenital alopecia (**Figure 1B**).

All patients remained nonbullous and exhibited persistent, non-resolving ichthyosis throughout follow-up, distinguishing their course from the self-improving collodion baby phenotype.

Additional appendageal involvement further illustrated the severity of the disease. All three patients in Family and P2.11 exhibited pronounced nail dystrophy, characterized by thickened, brittle, and ridged nails, often accompanied by subungual hyperkeratotic debris. Hair abnormalities were frequent: both P2.2 and P3.1 had total alopecia from birth, while the remaining patients demonstrated sparse, coarse hair with patchy areas of alopecia in regions of prominent hyperkeratosis, suggesting cicatricial involvement. Ocular complications were also present, notably in P1.2, who exhibited persistent bilateral ectropion beyond the neonatal period. These findings demonstrate a broad clinical spectrum of ARCI associated with *ALOX12B* variants, spanning LI and CIE phenotypes.

### Systemic and infectious manifestations

Patients from Family 1 and 2 demonstrated a high burden of recurrent cutaneous infections (**Table 2**). In Family 1, both P1.1 and P1.2 developed severe, recurrent bacterial and fungal skin infections, beginning after the age of three, primarily caused by *Staphylococcus aureus* and *Candida albicans*. These episodes frequently necessitated hospitalization and intravenous antibiotic therapy. Both patients were also diagnosed with severe, treatment-resistant asthma, requiring ongoing management with high-dose inhaled corticosteroids and bronchodilators at the last follow-up time. Notably, P1.2 had experienced two episodes of pneumonia, both requiring hospitalization and intravenous antibiotic therapy. In contrast, their younger sibling, P1.3, experienced only occasional, mild skin infections, which were successfully managed in the outpatient setting, and had no history of asthma or other allergic diseases.

**Table 2:** Infectious complications observed in ALOX12B-deficient patients.

Subject	Frequency of infections	Predominant site(s)	Causative organism(s)	Hospitalization required
P1.1	Recurrent	Skin	*S. aureus*, *C. albicans*	Yes (sepsis)
P1.2	Recurrent	Skin, respiratory tract	*S. aureus* *C. albicans* *H. influenzae*	Yes (pneumonia)
P1.3	Recurrent	Skin	Not available	No
P2.1	Recurrent	Skin	*S. aureus* *Candida* spp.	No
P2.2	Frequent (neonatal period + infancy)	Skin, blood	*S. aureus* *C.albicans* *P. aeruginosa*	Yes (pneumonia, sepsis)
P3	Only neonatal	Blood	Not available	Yes (neonatal sepsis)

Frequency of infections refers to recurrent episodes (>3 per year) or single severe systemic events. Microbial agents were identified by culture where available; otherwise, inferred from clinical course and response to antibiotics/antifungals. Hospitalization refers to infections requiring inpatient intravenous therapy or neonatal intensive care.

In Family 2, both affected siblings (P2.1 and P2.2) exhibited recurrent cutaneous infections, predominantly bacterial and fungal, requiring repeated courses of oral antibiotic therapy. P2.2 had a history of neonatal sepsis and pneumonia, requiring a 51-day admission to the neonatal intensive care unit (NICU). She was diagnosed with chronic lung disease as a sequela of neonatal acute respiratory distress syndrome and also suffered from severe asthma, requiring ongoing follow-up by pediatric pulmonology. Notably, between the ages of 5 months and 1 year, she subsequently experienced recurrent hospitalizations due to pneumonia and sepsis, including one admission to the pediatric intensive care unit (PICU) at 6 months of age. During acute respiratory exacerbations, she required positive airway pressure support. In addition, elevated liver enzyme levels and coagulation abnormalities were observed during both the initial hospitalization at 5 months and the PICU stay at 6 months. She underwent a comprehensive evaluation for potential underlying causes, including metabolic disorders, but no specific etiology was identified. These abnormalities were transient and resolved during the follow-up period. By contrast, her older brother P2.1 had no history of respiratory illness or organ dysfunction beyond the ichthyosis phenotype. None of the patients had any comorbidities or were receiving immunosuppressive medications that could predispose them to infections.

P3 had mostly mild systemic involvement: aside from a single episode of neonatal sepsis, there were no infections. However, P3’s neonatal course was complicated by an umbilical venous catheter–associated thrombosis in the first days of life. This thrombus was treated with therapeutic enoxaparin, with complete resolution.

### Immunological evaluation

In Family 1, all three siblings (P1.1, P1.2, and P1.3) exhibited markedly elevated total IgE levels exceeding 2500 IU/mL. P1.1 and P1.2 demonstrated polyclonal hypergammaglobulinemia with elevated IgG levels, but without significant eosinophilia. Functional humoral immunity demonstrated interindividual variability, with discordant serological responses to protein-based vaccines, ranging from protective to absent titers, despite complete vaccination records.

In Family 2, both siblings (P2.1 and P2.2) had normal serum IgE and immunoglobulin levels, with no evidence of antibody deficiency or systemic immune dysfunction. Vaccine-specific antibody titers were largely protective in both. Despite P2.2’s history of recurrent severe infections and multi-organ involvement, immunological evaluation revealed only subtle abnormalities, including an increased proportion of non-switched memory B cells and decreased switched memory B cells, while overall humoral and cellular immunity remained within normal limits. Although her IgG levels were below age-appropriate reference ranges during the first year of life, she mounted adequate vaccine responses, and her IgG levels normalized over time.

P3 was referred after an incidental finding of low serum IgG (350 mg/dL, below age-adjusted normal) discovered during routine labs. Her total IgE was elevated (1800 IU/mL). IgA and IgM were within normal range. Lymphocyte subset analysis was overall unremarkable, except for a reduction in naïve B cells and non-switched memory B cells, and vaccination titers (hepatitis B, mumps, rubella) were protective. Notably, she had no history of recurrent infections despite low IgG levels.

**Figure 2:**
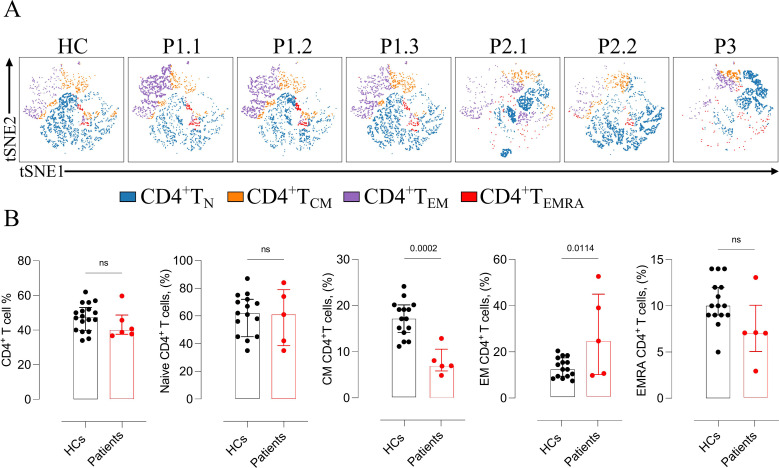
Altered CD4^+^ T-cell subset distribution in ALOX12B-deficient patients and comparison with healthy controls. **(A)** Representative t-SNE plots illustrating the distribution of CD4^+^ T-cell subsets — naïve, central memory (CM), effector memory (EM), and terminally differentiated effector memory RA^+^ (EMRA) — of patients. **(B)** Quantitative comparison of total, naïve, and memory (CM, EM, EMRA) CD4^+^ T-cell subsets between ALOX12B-deficient patients and age-matched healthy controls (HCs). Data are presented as individual values, with black dots representing healthy controls and red dots representing patients. Error bars indicate median with interquartile range. Statistical analysis was performed using the Mann–Whitney U test, ns: non-significant.

Comprehensive lymphocyte immunophenotyping was performed and interpreted in comparison with age-matched HCs. Total T- and B-cell counts were within normal reference ranges in all patients. However, P2.2 displayed an increased proportion of non-switched memory B cells, and P3 exhibited reduced frequencies of both naïve and non-switched memory B cells, without evidence of overt humoral immunodeficiency. Within the CD4^+^ T-cell compartment, abnormalities were identified at the level of the memory subset, with proportions deviating from age-adjusted reference ranges and subsequently confirmed through direct comparison with HCs. Total and naïve CD4^+^ T-cell frequencies were preserved (p = 0.3 and p = 0.8, respectively). In contrast, central memory CD4^+^ T cells were significantly decreased (p = 0.0002) and effector memory CD4^+^ T cells were increased (p = 0.011), while terminally differentiated effector memory RA^+^ CD4^+^ T cells remained comparable (p = 0.06) (**Figures 2A, B**). In contrast, the distribution of total, naïve, and memory CD8^+^ T-cell subsets were indistinguishable from that of HCs (p = 0.12, p = 0.27, p = 0.34, respectively).

Allergen-specific IgE testing was negative in all patients. None fulfilled diagnostic criteria for a defined PID, such as HIES syndrome or combined immunodeficiency. The detailed immunological findings of the patients are summarized in **Table 3**.

**Table 3:** Immunological findings of ALOX12B-deficient patients.

Patient	P1.1	P1.2	P1.3	P2.1	P2.2	P3
Leukocyte count (/mm3)	7320	7720	8000	13100	10500	8500
- Absolute lymphocyte count	2340	1820	3410	6500	6200	4800
- Absolute neutrophil count	4140	5000	3880	5300	3400	3400
- Absolute eosinophil count	22	30	20	360	320	60
Immunoglobulins						
IgA (mg/dL)	147 (139– 2378)	230 (108–447)	100 (678–383)	72 (26–296)	29 (13–72)	35 (30–307)
IgG (mg/dL)	**2200 (↑)** **(913**– 1884)	**2750 (↑)** **(984**–1958)	1290 (764 –2134)	666 (604–1941)	514 (294–1165)	**350 (↓)** **(605**– 1430)
IgM (mg/dL)	160 (88–322)	**320 (↑)** **(83**–282)	92 (69–387)	82 (71–235)	42 (33–154)	**40 (↓)** **(66**– 228)
IgE (IU/ml)	**>2500 (↑)**	**>2500 (↑)**	**>2500 (↑)**	32	6.5	**1800 (↑)**
Vaccine responses						
Anti-Hbs	positive	negative	positive	positive	positive	positive
Anti-Mumps	negative	negative	positive	positive	positive	positive
Anti-Rubeola	positive	positive	positive	positive	positive	positive
Anti-Pneumococcus	negative	negative	positive	NA	NA	NA
Lymphocyte subsets						
CD3+ T cell count (/mm3)	1497 (1025–2793)	1664 (1033–3305)	2782 (1213–4128)	4030 (1200–4706)	3472 (1492–6385)	2736 (1982–7372)
CD3+CD4+ T cell count (/mm3)	889 (621–1631)	1092 (504–1771)	1568 (607–2111)	2405 (458–2755)	2542 (909–4523)	1872 (1211–4686)
CD3+CD8+ T cell count (/mm3)	585 (269–1255)	527 (381–1312)	1091 (380–2083)	1300 (165–1878)	868 (254–2123)	768 (567–2494)
CD19+ B cell count (/mm3)	444 (87–540)	127 (94–792)	238 (197–867)	1300 (205–1341)	2108 (237–2564)	1632 (526–3126)
CD16 + 56+ NK cell count (/mm3)	351 (100–640)	**18 (↓)** **(94**–1174)	341 (111–963)	715 (88–1393)	434 (101–1633)	173 (105–1461)
CD19+CD27-IgD+ B cells, (%)	60 (36–85)	54 (46–92)	56 (55–90)	NA	85 (78–96)	94 (69–97)
CD19+CD27+IgD+ B cells, (%)	12 (8–38)	8 (5–28)	9 (6–28)	NA	**11 (↑)** (0.9-9)	**2 (↓)** **(3**–19)
CD19+CD27+IgD- B cells, (%)	17 (8–44)	16 (6–35)	17 (7–31)	NA	**0.34 (↓)** (0.8-8.7)	**1(↓)** **(2**–17)
CD21low CD38low activated B, (%)	4 (1–14)	7 (1–15)	10 (2–13)	NA	4 (0.5-5)	2 (1–6)
CD4+ CD45RA+ CD31+ T cells, (%)	32 (7–80)	44 (28–61)	58 (37–73)	NA	65 (57–91)	62 (60–83)
CD4+ CD45RA+ CCR7+ T cells, (%)	35 (16–87)	42 (31–70)	61 (40–79)	NA	84 (64–97)	74 (52–98)
CD4 + CD45RA- CCR7+ T cells, (%)	**7 (↓)** **(26**–72)	**7 (↓)** **(26**–53)	**8 (↓)** **(17**–53)	NA	5 (2–23)	13 (8–38)
CD4+ CD45RA- CCR7- T cells, (%)	**52 (↑)** **(3**–25)	**38 (↑**) **(4**–23)	**24 (↑)** **(2**–14)	NA	5 (0.2-9)	10 (1–11)
CD4+ CD45RA+ CCR7- T cells, (%)	7 (1–44)	13 (1–21)	7 (1–43)	NA	7 (0.06-50)	3 (1–55)
CD8+ CD45RA+ CCR7+ T cells, (%)	40 (11–92)	56 (17–72)	45 (22–67)	NA	86 (28–95)	85 (14–90)
CD8+ CD45RA- CCR7+ T cells, (%)	2 (1–28)	5 (2–14)	2 (2–10)	NA	4 (0.3-6)	4 (1–8)
CD8+ CD45RA- CCR7- T cells, (%)	32 (7–50)	24 (10–45)	18 (7–41)	NA	3 (1–35)	5 (3–40)
CD8+ CD45RA+ CCR7- T cells, (%)	28 (13–78)	15 (15–77)	35 (18–74)	NA	7 (2–55)	6 (13–69)

CD, Cluster Differentiated; CCR7, Chemokine receptor 7; Ig, Immunoglobulin; NA, Not available; NK, Natural Killer; TCR, T Cell Receptor; ↑, Above age-matched reference range; ↓, Below age-matched reference range. Values outside the age-specific reference ranges are indicated in bold.

### Genetic findings

**Figure 3:**
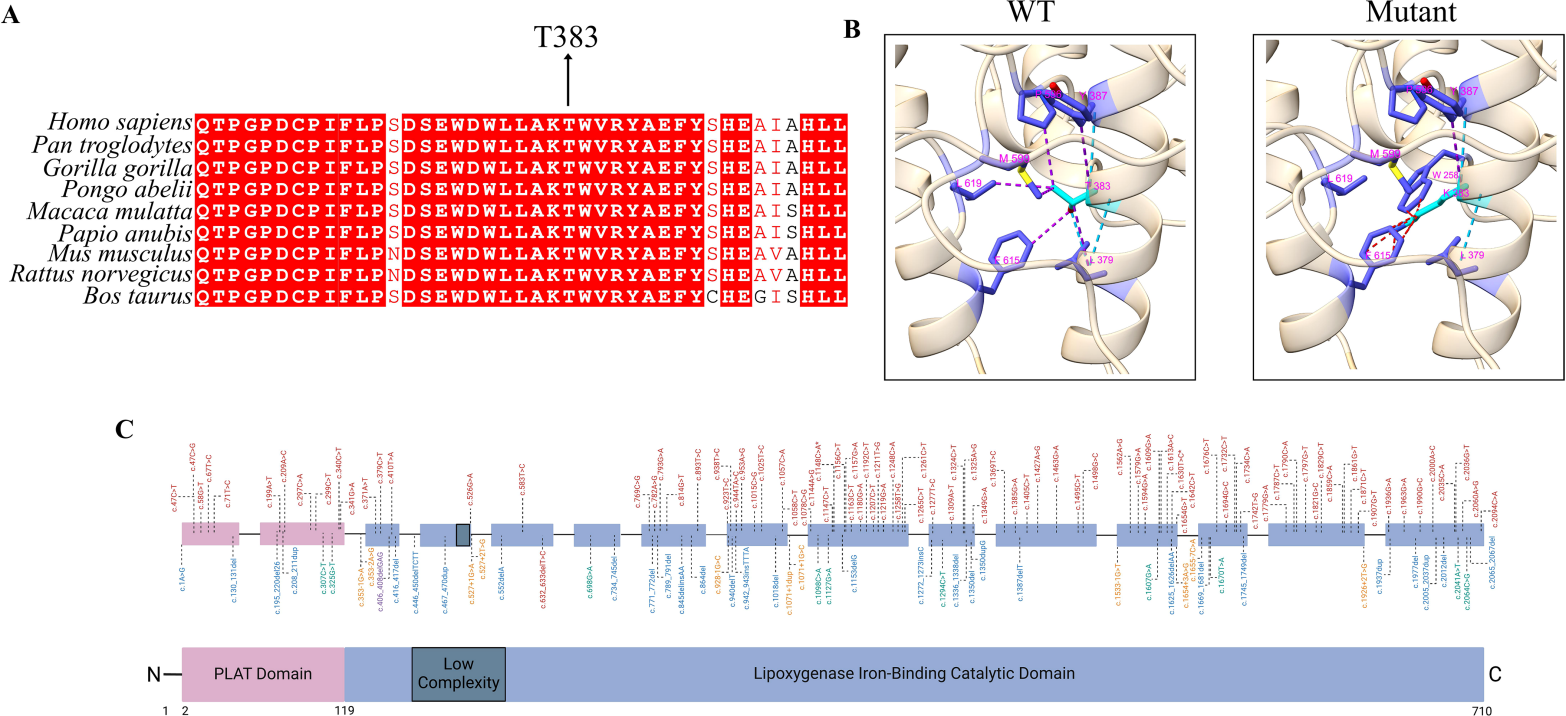
Evolutionary conservation, structural impact, and mutational landscape of *ALOX12B* gene variants. **(A)** Multiple sequence alignment (MSA) showing the interspecies conservation of the ALOX12B protein region spanning amino acids 360–400. The threonine residue at position 383 (highlighted) is highly conserved across species, underscoring its structural and functional importance within the catalytic domain. **(B)** Structural comparison of the wild-type and mutant (p.Thr383Lys) ALOX12B proteins using ChimeraX v1.10.1. Light blue lines indicate hydrogen bonds, purple lines represent contacts, and red lines denote steric clashes between residues. **(C)** Schematic representation of the ALOX12B (12R-lipoxygenase) protein structure and distribution of pathogenic variants. The ALOX12B protein comprises an N-terminal PLAT (polycystin-1, lipoxygenase, α-toxin) domain (pink), a central low-complexity region (dark gray), and a C-terminal iron-binding lipoxygenase catalytic domain (blue), based on transcript NM_001139.3. Pathogenic variants previously associated with autosomal recessive congenital ichthyosis (ARCI) are plotted above the schematic, color-coded by mutation type: red for missense mutations, blue for frameshift mutations, green for nonsense mutations, and yellow for non-coding mutations. The novel p.Thr383Lys and recurrent p.Cys544Arg missense variants identified in this study are indicated with asterisks (*).

In Family 1, all three affected siblings (P1.1, P1.2, and P1.3) carried the homozygous missense variant c.1148C>A (p.Thr383Lys) in the *ALOX12B* gene. This variant is absent from population databases such as gnomAD and ExAC, indicating that it is not a common polymorphism. In silico pathogenicity prediction tools consistently support a deleterious effect: SIFT and AlphaMissense both classify the variant as “damaging,” PolyPhen-2 predicts it as “probably damaging,” and it displays a high CADD score, collectively suggesting a strong likelihood of functional disruption. The threonine residue at position 383 is highly conserved across species and is located within the catalytic domain of the 12R-lipoxygenase enzyme, emphasizing its structural and functional significance (**Figure 3A****).** Structural modeling of the wild-type (WT) enzyme revealed that Thr383 forms three hydrogen bonds with Leu379 and Tyr387. Tyr387, in turn, engages in six stabilizing contacts with Leu379, Phe615, Leu619, Met599, and Pro596. In the mutant form (T383K), two of these hydrogen bonds and five of the nonbonded contacts are disrupted, leading to a loss of intramolecular stabilization. Furthermore, the introduction of Lys383 results in 11 steric clashes with Trp258 and Phe615, indicating substantial local structural strain and conformational perturbation (**Figure 3B****).**

Physicochemical analysis shows that the mutant residue is larger, positively charged, and less hydrophobic than the WT threonine, which may interfere with optimal residue packing and intra-domain interactions. Consistently, DDMut analysis (21) predicted a stability change (ΔΔG^Stability wt->mt^) of –1.78 kcal/mol, supporting a significant destabilizing effect on the overall protein structure.

Collectively, the absence of this variant in population databases, the concordant deleterious computational predictions, the high evolutionary conservation of the affected residue, and the predicted destabilization of the catalytic domain together support its classification as likely pathogenic according to ACMG guidelines (**Table 1**).

In Families 2 and 3, the affected individuals (P2.1, P2.2, and P3) were homozygous for the c.1630T>C variant, resulting in the p.Cys544Arg substitution (22). This variant has been previously reported in the literature (Variation ID: 995485) with a classification of ‘pathogenic’ (**Table 1**; **Figure 3C**). The recurrence of this variant in unrelated families with similar severe phenotypes further supports its pathogenicity. No other potentially disease-causing variants were identified in known PID genes or in other genes previously associated with ARCI.

### Treatment and clinical response

All six patients received standard therapies for ARCI, including daily emollients, keratolytic agents, topical corticosteroids during inflammatory exacerbations, and oral retinoids in selected cases. However, treatment responses were variable across individuals and families.

P1.1 and P1.2 had severe, treatment-refractory ichthyosis. Despite consistent adherence to topical and systemic therapies, including courses of oral acitretin, no significant improvement was observed in their skin lesions. Both patients were later initiated on monthly intravenous immunoglobulin (IVIG) therapy at a standard dose of 0.5 g/kg, primarily due to recurrent, severe bacterial and fungal skin infections. Following IVIG initiation, they experienced a marked reduction in the frequency and severity of infections, indicating a potential protective effect. However, IVIG did not significantly improve ichthyotic skin findings. Their younger sibling, P1.3, with milder skin involvement and less frequent infections, was managed effectively with topical treatments alone.

P2.1 and P2.2 received systemic retinoic acid in addition to standard topical therapies. While P2.1 showed clinical improvement in scaling and erythema, P2.2 exhibited a minimal response, suggesting interindividual variability in retinoid efficacy. P3 was also treated with oral retinoic acid and demonstrated notable improvement in scaling and erythema (**Figure 1B**).

## Discussion

This case series highlights the complex phenotypic and immunologic spectrum associated with ALOX12B-related ARCI. Although traditionally considered a dermatologic condition, our findings demonstrate that *ALOX12B* variants may also be associated with systemic manifestations and immune dysregulation, emphasizing the importance of a multidisciplinary diagnostic and therapeutic approach. While previously published cohorts have reported limited genotype–phenotype correlations in ARCI, with *ALOX12B* variants typically linked to mild-to-moderate ichthyosis or self-improving collodion baby phenotypes, our data expand this clinical spectrum (1, 3, 8, 23)​. Notably, we observed that identical pathogenic variants can give rise to highly divergent disease severity and progression, even among affected individuals within the same family.

Importantly, patients in our cohort showed immune abnormalities, most notably significantly elevated serum IgE levels and, in some cases, increased vulnerability to infections, that appear to extend beyond what is usually reported in classic ARCI. To our knowledge, such extreme IgE elevation has not been previously described in ALOX12B-related ARCI. Nevertheless, none of the affected individuals fulfilled the clinical or immunologic criteria for classical PID or HIES; most patients showed normal vaccine responses and typical lymphocyte subset profiles (24, 25). The only consistent immune difference observed was a specific change in the CD4^+^ T-cell memory compartment, with decreased central memory and increased effector memory CD4^+^ T cells. These results indicate a pattern more aligned with barrier-related immune dysregulation rather than a PID, as chronic epidermal barrier disruption, such as that observed in ichthyosis, is increasingly recognized to drive secondary systemic immune activation through sustained antigen exposure and low-grade inflammation (4). Similar to filaggrin-deficient atopic dermatitis, persistent barrier failure may facilitate the transcutaneous entry of microbial or environmental antigens, promoting Th2 and Th17 polarization and elevated IgE production ​ (26). Recent transcriptomic studies confirm this paradigm by demonstrating a strong IL-17/Th17 signature in ichthyotic skin, with correlations between IL-17 expression and disease severity in both skin and serum ​ (27, 28). Moreover, persistent skin inflammation may secondarily skew immunity toward atopy: studies in barrier-deficient skin, such as atopic dermatitis, show increased thymic stromal lymphopoietin and Th2 cytokines that promote IgE class switching (6, 29, 30)​. Although we did not directly assess cytokine profiles, the marked elevation of IgE and the presence of asthma in some patients suggest a shift toward systemic atopy, potentially secondary to cutaneous immune stimulation. We therefore hypothesize that the extreme IgE levels observed in Families 1 and 3 may result from ongoing immune activation through a “leaky skin barrier” acting as a sensitization site. However, the absence of eosinophilia and allergen-specific IgE in our patients suggests that this immune profile may differ from classical atopy and instead involve innate immune activation through lipid-mediated pathways (e.g., IL-1β/IL-23 axis). Further studies assessing cytokine signatures in ALOX12B-deficient skin are needed to delineate these mechanisms.

While ALOX12B-ARCI shares surface phenotypic features with syndromic ichthyoses, such as Netherton syndrome or STAT3 HIES, its immunological footprint appears milder, incomplete, and most consistent with secondary immune dysregulation. This emphasizes the importance of integrated dermatologic-immunologic evaluation in these patients, as summarized in **Table 4**.

**Table 4:** Comparative features of ALOX12B-ARCI and cutaneous disorders associated with immune deficiency.

Syndrome	Gene	Skin phenotype	Atopic features	Immunologic features	Infection profile	Associated features
ALOX12B-ARCI	*ALOX12B*	Lamellar ichthyosis, collodion membrane, nail dystrophy, alopecia, CMC	Asthma, allergic rhinitis	Mild to moderate ↑ IgE, hypo-/hypergammaglobulinemia, normal lymphocyte subsets, selective vaccine response deficits in some cases	Recurrent bacterial/fungal skin infections, pneumonia, bacterial/fungal sepsis, neonatal sepsis	Hypoplastic fingers and toes, ectropion, steroid-resistant nephrotic syndrome (rare)
Netherton Syndrome (34)	*SPINK5*	Generalized erythroderma, ichthyosis linearis circumflexa, trichorrhexis invaginata, congenital lamellar ichthyosis	Asthma, urticaria, angioedema, food allergies	↑ IgE, eosinophilia, hypogammaglobulinemia, ↓ memory B cells, impaired vaccine responses, ↓ naïve CD4+ T cells, ↓ NK function	Recurrent skin, sinopulmonary infections and sepsis	Enteropathy with villous atrophy, intestinal atresia, developmental delay
SAM Syndrome (35)	*DSG1*	Generalized congenital erythroderma ichthyosis, psoriasiform dermatitis, hypotrichosis	Food allergies, eosinophilic esophagitis	↑ IgE, eosinophilia, ↓ B-cell count, ↓ T-cell proliferation	Recurrent skin and respiratory infections, sepsis	NMR, VSD, PS, GOR, malabsorption, microcephaly
STAT3-HIES (36)	*STAT3*	Severe eczematous dermatitis, recurrent cold abscesses, CMC	Atopic dermatitis	↑ IgE, eosinophilia, ↓ memory B cells, impaired vaccine responses, ↓ Th17 cells	Recurrent sinopulmonary infections, skin abscesses, recurrent fungal infections	Coarse facies, prominent forehead, mild prognathism, hypertelorism, broad nose, high-arched palate, retained primary teeth, joint hyperextensibility, recurrent fractures, scoliosis, vascular aneurysm.
DOCK8 Deficiency (37)	*DOCK8*	Severe eczematous dermatitis, molluscum contagiosum, warts, CMC, recurrent herpes infections	Atopic dermatitis, asthma, food allergies	↑ IgE, eosinophilia, ↓ IgM, T- and B-cell lymphopenia, impaired vaccine responses, ↓ naïve CD4+ T cells, ↓ Treg frequency and function, ↓ NK cell activity	Recurrent bacterial/fungal/viral skin, sinopulmonary, deep tissue infections, sepsis, pneumatocele	Autoimmunity, increased risk of malignancies, vascular aneurysm, vasculitis
PGM3 Deficiency (38)	*PGM3*	Eczematous dermatitis, cutaneous vasculitis, erythema multiforme major, skin abscesses	Atopic dermatitis, asthma, allergic rhinitis, food allergies	↑ IgE, normal/high IgA and IgG, T- and B-cell lymphopenia, ↓ T-cell proliferation, SCID-like phenotype	Recurrent bacterial and viral skin/sinopulmonary infections, sepsis	Sensorineural hearing loss, wide nostrils, high arched palate, prominent lips, scoliosis, NMR, hypotonia

ARCI, Autosomal Recessive Congenital Ichthyosis; CMC, Chronic Mucocutaneous Candidiasis; GOR, Gastroesophageal Reflux; IgE, Immunoglobulin E; NK, Natural Killer; NMR, Neuromotor Growth Retardation; PS, Pulmonic Stenosis; SCID, severe combined immunodeficiency; STAT3-HIES, Hyper-IgE Syndrome due to *STAT3* mutations; Th17, T-helper 17; Treg, T regulatory; VSD, Ventricular Septal Defect.

Therapeutically, our experience indicates that standard keratolytic and emollient regimens were variably effective and often insufficient in managing severe ichthyoses. Notably, two siblings (P1.1, P1.2) with recurrent infections and hyper-IgE received standard-dose monthly IVIG treatment as a preventive measure against infections and related complications. This intervention was highly effective in reducing the frequency of infections; however, no significant improvement in skin manifestations was observed. Previous studies have demonstrated that high-dose immunoglobulin replacement therapy (IgRT) may benefit Th2/Th17-mediated dermatoses, such as atopic dermatitis, through various immunomodulatory mechanisms, including pathogen and autoantibody neutralization, anti-inflammatory Fc-mediated signaling, and modulation of cytokine networks (31, 32). For instance, a controlled trial in patients with severe atopic dermatitis showed that monthly IVIG infusions at 2 g/kg reduced disease severity and lowered Th2 cytokine levels, such as interleukin-5 (33)​. Although our patients did not show skin improvement, the infection control observed underscores the potential of IVIG as an adjunctive therapy in ARCI patients with infectious complications. Further investigation is needed to clarify the role of IgRT in ichthyosis, particularly whether higher or sustained dosing regimens could offer benefits beyond infection control, including effects on serum IgE levels and T-cell polarization.

Our findings underscore the importance of multidisciplinary care in genodermatoses from an immunological perspective. Consultation with Allergy/Immunology specialists is also beneficial, as patients with hyper-IgE or antibody deficiencies may require IgRT or prophylactic antibiotics as part of their preventive management. ARCI with immune involvement demands coordination between dermatologists, immunologists, ophthalmologists, pulmonologists, and geneticists. Such a team approach can optimize skin care while anticipating and managing systemic complications, ultimately improving outcomes for these complex patients.

This study is limited by its small sample size, which restricts the generalizability of the findings. While identifying recurrent immunologic abnormalities, including elevated IgE, antibody dysfunction, and increased susceptibility to infections, is compelling, the absence of functional immunologic assays (e.g., cytokine profiling, T-cell polarization studies) hinders the drawing of mechanistic conclusions. Additionally, while two distinct *ALOX12B* variants were characterized, no *in vitro* enzymatic or lipidomic validation was performed to confirm their functional consequences. Finally, the clinical heterogeneity observed, even among patients with the same genotype, suggests that unrecognized modifier genes or environmental factors may contribute to phenotype, which could not be addressed within the scope of this study.

## Conclusion

This case series expands the clinical spectrum of ALOX12B*-*associated ARCI by integrating dermatologic, immunologic, and genetic perspectives. We observed systemic immune perturbations in some patients, including recurrent infections, markedly elevated serum IgE, CD4^+^ T-cell memory skewing, and asthma, suggesting that chronic skin barrier disruption may contribute to secondary immune dysregulation. While these findings do not indicate a specific PID, they underscore the importance of immunologic vigilance and multidisciplinary management in ARCI. Practically, patients with ALOX12B-related ichthyosis should be monitored for their skin condition and signs of immunological imbalance. Early intervention in infections and the appropriate use of antibiotics or antifungals can improve outcomes. Adjunctive therapies, such as IgRT, may play a role in controlling infections in select cases.

## Data Availability

The original contributions presented in the study are included in the article/**Supplementary Material**. Further inquiries can be directed to the corresponding author.
