# Supplementary material for: Beyond the skin: immunological profiles and infectious complications in ALOX12B-associated autosomal recessive congenital ichthyosis
Source: Front Immunol. 2025 Nov 19;16:1662858. doi: 10.3389/fimmu.2025.1662858 (PMC12672872; doi:10.3389/fimmu.2025.1662858)
Supplement: Supplementary file 1 [file DataSheet1.docx]

**Supplementary File**

**Beyond the Skin: Immunological Profiles and Infectious Complications in *ALOX12B*-Associated Autosomal Recessive Congenital Ichthyosis**

**Supplementary materials and methods**

***Antibodies and flow cytometry***

To determine deep lymphocyte subsets, the following monoclonal antibodies (mAbs) were used: Fluorescein isothiocyanate (FITC)-conjugated CD3 (UCHT1, 1:50, BC, FRA), Allophycocyanin (APC)-conjugated CD3 (33-2A3, 1:50, Immunostep, Spain), APC-Alexa Fluor 700 (APC-A700) CD4 (13B8.2, 1:50, BC), FITC CD4 (HP2/6, 1:50, Immunostep), APC CD4 (HP2/6, 1:50, Immunostep), Krome Orange (KO) CD45 (J33, 1:50, BC), Alexa Fluor 750 (APC-A750) CD45RA (2H4DH11LDB9, 1:50, BC), Phycoerythrin (PE) CD197 (CCR7) (G043H7, 1:50, BC), Pycoerythrin-Cyanin 7 (PC7) CD8 (SFCI21Thy2D3, 1:50, BC), APC-A700 CD14 (RMO52, 1:50, BC), PE CD16 (3G8, 1:50, BC), Pycoerythrin-Cyanin 5.5 (PC5.5) CD56 (N901, 1:50, BC), APC-A750 CD19 (J3-119, 1:50, BC), PB CD20 (B9E9, 1:50, BC), PB CD21 (BL13, 1:50, BC), PB  CD31 (5.6E, 1:50, BC), PC5.5 CD38 (LS198-4-3, 1:50, BC), Phycoerythrin-Texas Red-x (ECD) CD45RO (UCHL1, 1:50, BC), FITC IgD (IA6-2, 1:50, BC).

For lymphocyte subset analysis, 100µl of whole blood was incubated with mAbs against surface markers for 20 minutes in the dark at room temperature. Red cells were lysed and washed before acquisition. All stained cells were acquired with a Navios EX cytometer (Beckman Coulter) and analyzed with Kaluza Analysis Software (Version 2.1).

Peripheral T cells were divided into subpopulations as follows: CD4^+^ naive T cells (CD4^+^ CD45RA^+^ CCR7^+^), central memory CD4^+^ T cells (CD4^+^ CD45RA^–^ CCR7^+^), effector memory CD4^+^ T cells (CD4^+^ CD45RA^-^ CCR7^-^), terminally differentiated effector memory CD4^+^ T cells (TEMRA, CD4^+^ CD45RA^+^ CCR7^-^), CD8^+^ naive T cells (CD8^+^ CD45RA^+^ CCR7^+^), central memory CD8^+^ T cells (CD8^+^ CD45RA^–^ CCR7^+^), effector memory CD8^+^ T cells (CD8^+^ CD45RA^–^ CCR7^–^), and TEMRA CD8^+^ T cells (CD8^+^ CD45RA^+^ CCR7^–^). Peripheral B cells were classified into four distinct populations: naive mature B cells (CD19^+^ CD27^–^ IgD^+^), non-class-switched memory B cells (CD19^+^ CD27^+^ IgD^+^), class-switched memory B cells (CD19^+^ CD27^+^ IgD^-^) and autoreactive CD21^low^CD38^low^ B cells.

***Genetic studies***

For Whole Exome Sequencing (WES), Genomic DNA was extracted from peripheral blood samples and 1$\text{μ}$g of DNA was used for exome capture using the IDT XGen exome target design or Agilent SureSelect Human All Exon. Generated libraries were sequenced using 75 bp paired-end sequencing on an Illumina NovaSeq-6000 and BGISeq-500 platform. Captured fragments were sequenced to achieve a minimum of 85% of the target bases covered at 20x or greater coverage. Sequence reads were mapped and aligned to the hg19 human genome reference assembly using Burrows-Wheeler Aligner (BWA)-mem and Single-Nucleotide Variants (SNV) and small Insertion/Deletion (INDEL) variants were called using Genome Analysis Toolkit (GATK). Variants were then annotated by VEP and ANNOVAR and filtered by GEMINI (GEnome MINIng) based on population allele frequency, potential genetic models, and variant deleterious predictions. In more detail, outputted variants were filtered by read depth (RD > 20), genotype quality score (GQ > 15), minor allele frequency (MAF < 0.01, as reference database 1K Genome and gnomAD employed), Combined Annotation Dependent Depletion (CADD) scores (CADD Phred > 5 or not available) and Variant Allele Frequency (VAF > 0.25). Along with these filters, also synonymous variants were filtered out. Finally, the possible effects of the variants on protein function were determined by using in-silico pathogenicity prediction tools such as SIFT, Polyphen, and MutationTaster, and the conservation of the mutated region was evaluated based on GERP scores.  Clinical and laboratory phenotypes, accompanied by the autosomal recessive inheritance model (due to consanguinity in all families), were prioritized during the evaluation. The pathogenicity of all disease-attributable gene variants was re-evaluated using the updated guideline for interpretation of molecular sequencing by the American College of Medical Genetics and Genomics (ACMG) criteria (1, 2).

Sanger sequencing was performed to confirm the mutation identified by the WES. Briefly, genomic DNA was amplified by a polymerase chain reaction, and amplimers were sequenced using the Big Dye Terminator v1.1 Cycle Sequencing Kit (Applied Biosystems; Life Technologies, Darmstadt, Germany) on an Applied Biosystems 3130 Genetic Analyzer.

**Supplementary patients’ descriptions**

**Family 1**

### **Patient (P) 1.1**

**A seventeen-year-old female patient born to consanguineous parents** presented with a history of progressive skin disease beginning after infancy. She was born without a collodion membrane and appeared phenotypically normal during the neonatal period. However, generalized scaling developed after the first year of life, eventually manifesting as **delayed-onset lamellar ichthyosis (LI)**. Her skin exhibited **thick, plate-like hyperkeratotic scales with minimal erythema**, and she developed **nail dystrophy** (brittle, thickened nails), without alopecia.

From age three onwards, she experienced **recurrent bacterial and fungal skin infections**, often requiring **hospitalization and systemic antibiotic treatment**. Additionally, she was diagnosed with **severe asthma and allergic rhinitis**, but **no sensitization to aeroallergens was detected**. **Blood cultures were positive for** Staphylococcus aureus **during episodes of infection.**

Immunological evaluation revealed **markedly elevated serum IgE (>2500 IU/mL)** and **polyclonal hypergammaglobulinemia (IgG: 2200 mg/dL).** Lymphocyte subset analysis was within normal limits. Despite being fully vaccinated, her **antibody responses to pneumococcus and mumps were non-protective,** suggesting impaired humoral memory.

Given her **history of frequent infections and hyper-IgE phenotype**, the patient was considered to fall within the spectrum of **hyper-IgE syndrome-like immunodysregulation**. However, no pathogenic variants were found in classical immunodeficiency genes. As a result, **monthly intravenous immunoglobulin (IVIG) therapy was initiated,** which **effectively reduced both cutaneous and systemic infections.** However**, IVIG had no appreciable impact on her cutaneous ichthyosis.**

Genetic testing identified a **novel homozygous missense mutation in the *ALOX12B* gene (c.1148C>A; p.Thr383Lys),** located in the lipoxygenase catalytic domain and not present in public databases. This variant is classified as likely pathogenic based on in silico analysis and evolutionary conservation. The patient continues to receive multidisciplinary follow-up for dermatologic and immunologic management.

### **P1.2**

This 14-year-old female patient, the younger sibling of P1.1, was born to consanguineous parents and presented with a similar dermatological phenotype of **delayed-onset LI.** She had **no collodion membrane at birth** but developed **generalized scaling** and **plate-like hyperkeratosis** during infancy. She exhibited **nail dystrophy, characterized by thickened and brittle nails, as well as bilateral persistent ectropion, which affected her** ocular comfort.

**Beginning at around age three to four,** she developed **recurrent cutaneous and lower respiratory tract infections,** including **two episodes of pneumonia,** both requiring **hospital admission and intravenous antibiotics.** Over time, she was also diagnosed with **severe asthma** and **allergic rhinitis. Growth failure and poor weight gain** were noted during follow-up, raising additional concerns about the underlying systemic impact.

Immunologic assessment revealed **markedly elevated IgE levels (>2500 IU/mL)** and **polyclonal IgG elevation,** with **lymphocyte subsets within normal limits. Antibody responses to routine vaccinations were variable**, with non-protective titers for mumps and pneumococcus despite documented immunization. There was no detectable allergen sensitization based on serum-specific IgE testing.

Because of her **recurrent infections, hyper-IgE phenotype,** and **suggestive features of immunodeficiency, monthly IVIG therapy was initiated**. Following IVIG treatment, **both skin and lung infections were effectively brought under control**, and **no new hospitalizations have occurred** since. However, **her ichthyotic skin disease and ocular findings (ectropion) persist,** with only partial response to standard dermatologic therapies.

Genetic testing identified a **novel homozygous missense variant in the *ALOX12B* gene (c.1148C>A; p.Thr383Lys),** identical to her two siblings, and located within the enzyme’s catalytic domain. This variant is predicted to be deleterious and has not been previously reported in public variant databases. She remains under multidisciplinary care involving dermatology, immunology, and pulmonology.

### **P1.3**

This eight-year-old male patient is the youngest sibling in Family 1, born to parents who are consanguineous. He presents with a milder phenotype of LI compared to his older sisters. He was not born with a collodion membrane but developed generalized scaling and hyperkeratosis during infancy. A dermatological examination revealed nail dystrophy, including thickened, brittle, and ridged nails, although his skin involvement was less extensive than that of his siblings.

Unlike P1.1 and P1.2, he **did not develop asthma, allergic rhinitis, or severe infections**. He experienced only **occasional mild skin infections**, all of which were successfully managed in the outpatient setting without hospitalization or systemic antibiotic therapy.

Immunologically, he had **markedly elevated serum IgE levels (> 2,500 IU/mL)**, similar to those of his siblings. However, his **IgG, IgA, and IgM levels were within normal ranges**, and **lymphocyte subset analysis showed no abnormalities**. Functional humoral immunity was preserved, with **generally protective responses to routine childhood vaccinations.**

Genetic analysis confirmed that he carried the **same novel homozygous missense variant in the ALOX12B gene (c.1148C>A; p.Thr383Lys), which had been** identified in his two older sisters. This variant lies within the catalytic domain of the lipoxygenase enzyme and is predicted to be damaging.

### **Family 2**

**P2.1**

A six-year-old male patient, born to consanguineous parents, is the older sibling in Family 2. **He was not born with a collodion membrane (CM)** but developed **generalized erythema and fine scaling** during the first months of life. His clinical presentation was consistent with **nonbullous congenital ichthyosiform erythroderma (CIE).** Compared to his sister (P2.2), his overall disease course was milder. He exhibited **no alopecia or nail dystrophy**, and there was **no evidence of systemic involvement.**

Throughout his early childhood, he experienced mild but recurrent skin infections, including episodes of bacterial and fungal infections. These infections were **managed successfully in the outpatient setting** and did not require hospitalization or intravenous treatment. **He had no history of asthma, allergic rhinitis, or other allergic manifestations.**

Immunologic evaluation revealed **normal levels of IgG, IgA, and IgM, as well** as **age-appropriate distributions of lymphocyte subsets. Serological responses to routine vaccinations,** including hepatitis B, mumps, and rubella, were **fully protective,** indicating preserved humoral immunity.

Genetic testing identified a **homozygous missense variant in the *ALOX12B* gene (c.1630T>C; p.Cys544Arg)**, which has been previously reported as pathogenic. The patient was started on **systemic retinoid therapy,** which resulted in **noticeable improvement in skin scaling and erythema**.

He continues to be followed in the dermatology clinic and remains clinically stable, with **no need for immunologic or pulmonary interventions.**

P2.2

A three-year-old female patient, the younger sibling of P2.1, was born to consanguineous parents. At birth, she presented with clinical features consistent with CIE, including CM , total congenital alopecia, and generalized erythroderma. Due to the severity of her skin involvement, she required admission to the neonatal intensive care unit (NICU) for the first 51 days of life, during which she developed extensive cutaneous infections and lower respiratory tract infections, necessitating intravenous antimicrobial therapy and intensive supportive care.

**Throughout infancy and early childhood, she developed chronic lung disease secondary to neonatal acute respiratory distress syndrome and was diagnosed with severe asthma, requiring regular follow-up by pediatric pulmonology. Between the ages of 5 months and 1 year, she experienced multiple hospitalizations for pneumonia and sepsis, including admission to the pediatric intensive care unit (PICU) at 6 months of age. During these episodes, she developed acute respiratory failure necessitating positive airway pressure support. Microbiological cultures revealed sepsis caused by** Pseudomonas aeruginosa**,** Staphylococcus aureus**, and** Candida albicans**. Thoracic computed tomography demonstrated bilateral subsegmental atelectasis and pleuroparenchymal band formations, particularly in the lower lobes, consistent with evolving structural pulmonary disease.**

**During one PICU stay, she developed a catheter-associated venous thrombosis, which was successfully managed with therapeutic anticoagulation. She also exhibited elevated liver enzymes and coagulopathy during both her initial hospitalization at 5 months and subsequent PICU admission at 6 months. A thorough diagnostic workup, including metabolic screening, failed to reveal an underlying etiology. These hepatic and coagulation abnormalities were transient and resolved during follow-up.**

From an immunologic perspective, she exhibited **persistently low IgG levels during the first two years of life**, for which **oral amoxicillin prophylaxis** was initiated. Following immune recovery, her **IgG levels normalized,** and prophylaxis was discontinued. Her most recent immunologic evaluation showed **normal levels of IgG, IgA, and IgM, protective vaccine-specific antibody titers**, and **normal lymphocyte subsets**, with **no evidence of a primary immunodeficiency.**

Genetic analysis identified a **homozygous pathogenic *ALOX12B* missense variant (c.1630T>C; p.Cys544Arg)**, previously described in association with severe ARCI phenotypes. Despite **systemic retinoid therapy**, her **cutaneous findings remain only partially responsive**. She continues to require **multidisciplinary care,** including dermatology, pulmonology, infectious disease, hematology, and clinical immunology.

**Family 3**

P3.1

A one-year-old female patient, born to consanguineous parents, is the only affected child in Family 3. She wa**s born with a CM, generalized erythroderma,** and **total congenital alopecia**, consistent with **CIE.** Due to the severity of her skin findings at birth, she was admitted to the **NICU** for initial supportive care and monitoring.

During the neonatal period, she developed **sepsis** and was also diagnosed with an **umbilical venous catheter–associated thrombus,** which was successfully treated with **therapeutic anticoagulation**. Since discharge from the NICU, she has remained **clinically stable** without further infectious complications.

Immunologic evaluation during infancy revealed **isolated hypogammaglobulinemia**, with **IgG measured at 350 mg/dL,** while **IgA and IgM levels were within normal limits**. She also had **markedly elevated total IgE (~1800 IU/mL).** **Lymphocyte subsets were within age-specific reference ranges**, and her **vaccine responses to hepatitis B, mumps, and rubella were protective,** indicating preserved functional humoral immunity. In the absence of recurrent infections or systemic features, her **hypogammaglobulinemia was interpreted as likely transient and not clinically significant.**

Genetic analysis revealed a **homozygous pathogenic missense variant in the *ALOX12B* gene (c.1630T>C; p.Cys544Arg),** the same variant identified in the affected siblings from Family 2. She was initiated on **oral retinoid therapy**, which resulted in **notable improvement in skin scaling and inflammation.** She continues to be followed in dermatology and immunology clinics for ongoing monitoring.

**Supplementary references**

1. Li Q, Wang K. InterVar: Clinical Interpretation of Genetic Variants by the 2015 ACMG-AMP Guidelines. Am J Hum Genet. 2017;100(2):267-80.

2. Richards S, Aziz N, Bale S, Bick D, Das S, Gastier-Foster J, et al. Standards and guidelines for the interpretation of sequence variants: a joint consensus recommendation of the American College of Medical Genetics and Genomics and the Association for Molecular Pathology. Genet Med. 2015;17(5):405-24.
